# Elevated sHLA-G plasma levels post chemotherapy combined with ILT-2 rs10416697C allele status of the sHLA-G-related receptor predict poorest disease outcome in early triple-negative breast cancer patients

**DOI:** 10.3389/fimmu.2023.1188030

**Published:** 2023-05-22

**Authors:** Oliver Hoffmann, Sebastian Wormland, Ann-Kathrin Bittner, Julian Hölzenbein, Esther Schwich, Sabine Schramm, Hana Rohn, Peter A. Horn, Rainer Kimmig, Sabine Kasimir-Bauer, Vera Rebmann

**Affiliations:** ^1^ Department of Gynecology and Obstetrics, University Hospital of Essen, Essen, Germany; ^2^ National Center for Tumor Diseases (NCT), NCT West, Essen, Germany; ^3^ Institute for Transfusion Medicine, University Hospital Essen, Essen, Germany; ^4^ Department of Infection Diseases, West German Centre of Infection Diseases, University Hospital of Essen, Essen, Germany

**Keywords:** triple-negative breast cancer, early breast cancer (EBC), biomarker, ILT-2 rs10416697C allele, sHLA-G, CTC (circulation tumor cells), HLA-G 3’ UTR

## Abstract

**Introduction:**

Triple negative breast cancer (TNBC) shows an aggressive growing and spreading behavior and has limited treatment options, often leading to inferior disease outcome. Therefore, surrogate markers are urgently needed to identify patients at high risk of recurrence and more importantly, to identify additional therapeutic targets enabling further treatment options. Based on the key role of the non-classical human leukocyte antigen G (HLA-G) and its related receptor immunoglobulin-like transcript receptor-2 (ILT-2) in immune evasion mechanisms of tumors, members of this ligand-receptor axis appear to be promising tool for both, defining risk groups and potential therapeutic targets.

**Materials and methods:**

To follow this, sHLA-G levels before and after chemotherapy (CT), HLA-G 3’ UTR haplotypes, and allele variations rs10416697 at the distal gene promoter region of ILT-2 were defined in healthy female controls and early TNBC patients. The results obtained were associated with clinical status, presence of circulating tumor cell (CTC) subtypes, and disease outcome of patients in terms of progression-free or overall survival.

**Results:**

sHLA-G plasma levels were increased in TNBC patients post-CT compared to levels of patients pre-CT or controls. High post-CT sHLA-G levels were associated with the development of distant metastases, the presence of ERCC1 or PIK3CA-CTC subtypes post-CT, and poorer disease outcome in uni- or multivariate analysis. HLA-G 3’ UTR genotypes did not influence disease outcome but ILT-2 rs10416697C allele was associated with AURKA-positive CTC and with adverse disease outcome by uni- and multivariate analysis. The prognostic value of the combined risk factors (high sHLA-G levels post-CT and ILT-2 rs10416697C allele carrier status) was an even better independent indicator for disease outcome in TNBC than the lymph nodal status pre-CT. This combination allowed the identification of patients with high risk of early progression/death with positive nodal status pre-CT or with non-pathological complete therapy response

**Conclusion:**

The results of this study highlight for the first time that the combination of high levels of sHLA-G post-CT with ILT-2 rs10416697C allele receptor status is a promising tool for the risk assessment of TNBC patients and support the concept to use HLA-G/ILT-2 ligand-receptor axis as therapeutic targets.

## 1 Introduction

Breast cancer (BC) is the most common cancer in women worldwide with almost 70.000 new diagnoses in Germany every year (1). In about 15-20% of the cases, early BC shows a triple negative (TNBC) behavior (2), defined by the lack of estrogen- and progesterone-receptor as well as the human epidermal growth receptor 2 (HER2) (3). Despite major improvement in diagnostic and therapy and an overall survival (OS) of 87% (1) the subgroup of early TNBC shows an aggressive behavior with a five-year distant recurrence of about 12%, resulting in an OS of about 82% (4, 5). Neoadjuvant chemotherapy (NACT) is the standard of care in early TNBC (6, 7), as response to therapy can be evaluated and therapy in the post-neoadjuvant setting can be adjusted. Pathological complete response (pCR) is a surrogate marker for improved progression-free survival (PFS) and OS (8, 9) in BC patients and therefore, a primary endpoint in clinical trials, leading to approval of new therapies. In the Keynote 522-trial, adding Pembrolizumab, a programmed cell death protein 1 (PD-1) inhibitor, in the neoadjuvant and post-neoadjuvant setting, showed a significant improvement in the pCR rate (10, 11) and event free survival (EFS) with a favorable trend for OS (12) in a subgroup of early TNBC patients with high risk of recurrence. Interestingly, in the GeparNUEVO trial, a better OS could be achieved in patients with a pCR after NACT in combination with Durvalumab (a programmed death-ligand receptor 1 inhibitor, PD-L1) compared to those achieving a pCR with no addition of immunotherapy to NACT (13). In case of a non-pCR after neoadjuvant chemotherapy, the addition of capecitabine post-neoadjuvant resulted in a prolonged PFS and OS rate (14). Recently, the PARP [Poly(ADP-ribose)-Polymerase]-Inhibitor Olaparib in the (postneo-) adjuvant setting showed a significantly improved EFS and distant disease free survival (15) as well as OS (16) in patients with gBRCAm and high risk of recurrence.

Nevertheless, since TNBC remains a biologically variable disease, treatment options are limited (17). Therefore, surrogate markers are urgently needed to identify patients at high risk of recurrence and in order to identify additional therapeutic targets enabling further treatment options.

In this context, non-classical human leukocyte antigen G (HLA-G) as immune-checkpoint (IC) molecule (18) appears to be a promising candidate because it is characterized by (i) restricted tissue distribution under physiological conditions, (ii) low degree of polymorphic variations in the coding region, (iii) and diverse immune modulating properties. HLA-G can exist as cell surface molecule or in soluble forms, either as released soluble membrane-free or vesicular-bound molecule. HLA-G and its soluble forms (sHLA-G) interact with immunoglobulin-like transcript (ILT) receptor-2 (LILRB1/CD85j), ILT-4 (LILRB2/CD85d) or killer inhibitory receptor (KIR) 2DL4 (18–26). Among all receptors, the ILT-2 receptor is the most abundant receptor in the periphery which is expressed on various lymphoid and myeloid cells of the adaptive or innate immune system (21, 27). Its interaction with HLA-G or sHLA-G results in the inhibition of diverse effector cell functions (18, 28). Consequently, an aberrant HLA-G/sHLA-G expression is considered to allow tumor immune escape (29). In this context, it is important to note that the HLA-G gene has significant variability of single nucleotide polymorphisms (SNP) in the 3’ untranslated region (3’ UTR), which form haplotypes that can affect HLA-G expression (30–32). In addition, it is reported that ILT-2 expression is regulated by SNP rs10416697C/G in the distal promoter region at position -14895 from the translational start (33).

Regarding BC, HLA-G expression has been detected in tumor tissues in about 66% of patients (34). High HLA-G tissue expression is often correlated with poorer disease status e.g. tumor size, nodal status, and stage as well as with inferior clinical outcome in terms of PFS and OS (34, 35). Interestingly, the genomic and immune profiling of a TNBC patient who progressed during combined NACT and PD-1/PD-L1 IC blockade, showed a low expression level of PD-1 receptor but high levels of HLA-G at the time of diagnosis (36). However, the access to tumor tissue is limited. To solve this problem, the use of blood as a liquid biopsy containing circulating tumor cells (CTC), immune relevant or tumor-associated molecules, especially soluble IC molecules such as sHLA-G, represents a promising platform to establish surrogate markers for the disease monitoring and prediction of outcome in cancer. For primary TNBC, the association of disease outcome with certain combinations of CTC subpopulations has already been identified by molecular characterization of CTC mRNA profiling (37). In non-metastatic locally advanced BC patients including a total of 18% TNBC patients, our study group recently demonstrated that high levels of vesicular-bound HLA-G before CT were associated with stem cell like CTC as well as with early disease progression (38). Moreover, certain HLA-G 3’ UTR haplotypes were found to predict therapy and disease outcome in that patient cohort (39).

However, for TNBC patients, limited information is available on the clinical and prognostic significance of sHLA-G and its association with CTC subpopulations. Furthermore, to the best of our knowledge, up to now, we are not aware of any study that analyses the clinical and prognostic significance of sHLA-G in the context of the genetic variations in the HLA-G 3’ UTR and in the ILT-2 distal promoter region at position -14570 (SNP rs10416697C/G). Considering the functional implication of the HLA-G/ILT-2 ligand-receptor axis as an IC in the tumor evolution, we hypothesized that sHLA-G, together with the genetic background of regulatory regions of HLA-G or ILT-2 expression, were relevant for risk assessment of TNBC patients. To prove this, (i) sHLA-G levels pre and post chemotherapy (CT), (ii) HLA-G 3’ UTR haplotypes, and (iii) allele variations of ILT-2 SNP rs10416697C/G were determined in association with clinical status, presence of CTC subpopulations and disease outcome of TNBC. The results of this study highlights for the first time that high levels of sHLA-G in combination with SNP rs10416697C ILT-2 receptor status are a potential surrogate marker for risk assessment of these patients.

## 2 Materials and methods

### 2.1 Characteristics of patients and healthy control panel

This retrospective trial was conducted at the Department of Gynecology and Obstetrics, at the University Hospital of Essen, Germany, evaluating 63 TNBC patients (56 pre-CT, and 50 post-CT; 42 paired patients), with first diagnosis of early BC between January 2013 and August 2018. EDTA samples of 16 age-matched healthy females (47 [35-62] years) presented the control panel. In addition, DNA samples of 163 female blood donors served as healthy control (HC) panel for HLA-G 3’ UTR and ILT-2 rs10416697 genotyping. Here, at time of blood sampling, the median age was 50 years ranging from 25 to 71 years.

#### 2.1.1 Eligibility criteria and response criteria

Patients enrolled in this trial were diagnosed with early TNBC and no metastasis at the time of first diagnosis. Patients with severe uncontrolled comorbidities or medical conditions and further malignancies at present or in the patient history were excluded. At the time of primary diagnosis and after NACT, if applicable, blood samples were obtained after written informed consent from all subjects [protocols used were approved by the clinical ethic committee of the University Hospital Essen (05/2856)]. Patients were treated according to current guidelines (6) including NACT and ACT (adjuvant chemotherapy) including anthracyclines, taxanes, cyclophosphamide, carbo- and cisplatin, myocet and gemcitabine as well as radiotherapy. Four patients received the PARP-inhibitor Olaparib in the GeparOla trial. Of the 64 patients included in our trial, one patient did not receive chemotherapy, four patients received chemotherapy in the adjuvant and 59 in the neoadjuvant setting. Pathological non-response was defined as regression 0 according to Sinn (40), pathological partial response (pPR) was defined as regression 1-3 according to Sinn, pCR was defined as regression 4 according to Sinn.

Patient characteristics before and after CT are documented in **Tables 1**; **2.** The aggressive behavior of TNBC was reflected by cT2 tumors in more than 50% of the patients, node positivity at the time of first diagnosis in about 31% of the cases, grade 3 tumors in almost 80% of the patients and the majority showed a Ki67 above 30%. 45.3% of the patients achieved a pCR after CT and 40.6% a pPR.

**Table 1:** Patients’ characteristics and their association of pre and post sHLA-G levels of TNBC patients.

Parameter	Pre-CT HLA-G (ng/ml)				Post-CT HLA-G (ng/ml)			
	n	Med.	Min	Max	n	Med.	Min	Max
Age (years)								
>60	15	10.60	3.44	73.50	14	14.22	5.10	43.41
<60	41	7.90	2.23	65.23	36	10.88	3.79	64.71
Menopausal Status								
Premenopausal	13	6.94	2.23	18.28	12	9.08	5.60	24.25
Perimenopausal	10	6.79	3.47	16.08	10	14.34	3.81	25.34
Postmenopausal	33	8.96	2.75	73.50	28	11.50	3.79	64.71
Histology								
Ductal	37	9.35	2.70	73.50	35	12.36	3.81	64.71
Lobular	1	12.67	12.67	12.67	1	18.28	18.28	18.28
Others	15	5.34	2.23	57.62	11	7.37	3.79	27.41
Unknown	3	10.60	7.28	14.61	3	16.38	3.81	20.67
Tumor grading								
I	0				0	.	.	.
II	12	8.20	4.28	41.28	9	14.96	3.81	25.34
III	43	8.59	2.23	73.50	41	10.82	3.79	64.71
unknown	1	57.62	57.62	57.62	0			
Ki 67								
0-10%	2	8.44	7.28	9.60	3	14.96	3.81	17.52
11-30%	6	7.78	4.28	13.92	4	10.77	3.81	17.02
>30%	36	8.58	2.23	73.50	35	10.65	3.79	64.71
unknown	12	10.38	3.92	57.62	8	13.65	6.02	43.41
Tumor size at diagnosis (c/pT)								
c/p T1a-c	23	7.40	2.70	22.26	19	12.22	3.79	64.71
c/p T2	28	8.68	2.23	65.23	27	10.65	3.81	25.34
c/p T3	3	10.12	5.34	73.50	3	13.05	7.51	24.25
c/p T4	2	13.99	12.51	15.47	1	14.97	14.97	14.97
Tumor size after CT (ypT)								
ypT0	24	8.68	2.23	57.62	24	10.74	3.79	64.71
ypT1	14	7.65	2.70	18.28	16	10.18	3.81	43.41
ypT2	11	10.60	3.44	73.50	8	15.68	6.77	25.34
ypT3	1	4.89	4.89	4.89	1	7.37	7.37	7.37
ypT4	1	5.24	5.24	5.24	1	26.46	26.46	26.46
unknown	5	9.11	3.34	12.51	0			
Nodal Status at diagnosis (c/pN)								
Node-negative (c/pN-)	38	8.58	2.23	73.50	35	10.10	3.79	43.41
Node-positive (c/pN+)	18	9.74	3.44	65.23	15	14.96	6.77	64.71
Nodal status after CT (ypN)								
Node-negative (ypN-)	3	12.60	4.28	13.27	4	9.16	3.81	15.39
Node-positive (ypN+)	2	40.35	15.47	65.23	0			
unknown	51	8.56	2.23	73.50	46	12.28	3.79	64.71
Chemotherapy								
Neoadjuvant	51	8.59	2.23	73.50	50	11.58	3.79	64.71
Adjuvant	4	6.52	3.34	10.97	0			
unknown	1	12.51	12.51	12.51	0			
Pathological response								
Complete response	25	8.59	25	8.59	25	10.82	3.79	64.71
Partial response	22	9.87	22	9.87	22	12.71	3.81	43.41
No response	4	5.12	4	5.12	3	7.37	6.77	17.16
unknown	5	9.11	5	9.11	0			
Distant metastases								
yes	8	6.74	3.34	65.23	***6***	***17.90***	***6.77***	***64.71***
no	47	8.76	2.23	73.50	***4***	***10.74***	***3.79***	***43.41***
unknown	1	12.51	12.51	12.51	0			
Recurrence (5y-PFS)								
Alive	45	8.96	2.23	73.50	42	10.74	3.79	43.41
Relapsed	10	7.00	3.34	65.23	8	17.34	3.81	64.71
unknown	1	12.51	12.51	12.51	0			
Overall survival (5y OS)								
Alive	47	8.76	2.23	73.50	***44***	***10.74***	***3.79***	***43.41***
Dead	8	6.74	3.34	65.23	***6***	***17.90***	***6.77***	***64.71***
unknown	1	12.51	12.51	12.51	0			

CT, chemotherapy; Med., Median; Min, Minimum; Max, Maximum; c, clinical; p, pathological; y, after neoadjuvant chemotherapy; statistical significance was tested by Mann-Whitney test; levels in bold italic indicate a significant difference *p<0.05.

**Table 2:** Patients’ characteristics and their association to ILT-2 rs10416697 C allele carrier status.

Parameter	Total (%)	^a^ILT-2 rs10416697 (n=63)		p-value
	n=64	C pos (n=29)	C neg (n=34)	
Age (years)				
>60	19 (29.7)	9	10 (29.1)	n.s.
<60	45 (70.3)	20 (69.0)	24 (70.6)	
Menopausal Status				
Premenopausal	17 (26.6)	6	11	n.s.
Perimenopausal	11 (17.2)	5	6	
Postmenopausal	36 (56.3)	18	17	
Histology				
Ductal	44 (68.8)	20	23	n.s.
Lobular	1 (1.7)	0	1	
Others	15 (23.4)	7	8	
unknown	4 (6.3)	2	2	
Tumor grading				
I	0	0	0	n.s.
II	12 (18.8)	6	5	
III	51 (79.7)	22	29	
unknown	1 (1.6)	1	0	
Ki 67				
0-10%	3 (4,7)	1	2	n.s.
11-30%	7 (10.9)	2	4	
>30%	41 (64.1)	23	18	
unknown	13 (20.3)	3	10	
Tumor size at diagnosis (c/pT)				
c/pT1a-c	23 (35.9)	11	11	n.s.
c/pT2	34 (53.1)	15	19	
c/pT3	4 (6.3)	2	2	
c/pT4	3 (4.7)	1	2	
Tumor size after NACT (ypT)				
ypT0	28 (43.7)	14	14	n.s.
ypT1	17 (26.6)	7	10	
ypT2	12 (18.7)	7	5	
ypT3	1 (1.6)	0	1	
ypT4	1 (1.6)	0	1	
unknown	5 (7.8)	14	14	
Nodal Status at diagnosis (c/pN)				
Node-negative (c/pN-)	44 (68.8)	23	20	n.s.
Node-positive (c/pN+)	20 (31.3)	6	14	
Nodal status after NACT (ycN)				
Node-negative (ycN-)	5 (7.9)	0	5	n.s.
Node-positive (ycN+)	2 (3.1)	1	1	
unknown	56 (88.0)	28	27	
Chemotherapy				
Neoadjuvant	59 (93.6)	28	31	n.s.
Adjuvant	4 (6.3)	1	2	
unknown	1 (1.6)	1	0	
Pathological response				
Complete response	29 (45.3)	14	15	n.s.
Partial response	26 (40.6)	12	14	
No response	4 (6.4)	2	2	
unknown	5 (7.8)	1	3	
Distant metastases				
Yes	9 (14.1)	7	2	***0.044***
No	54 (84.4)	22	31	
unknown	1 (1.6)	0	1	
Recurrence (5y-PFS)				
Alive	52 (81.3)	21	30	0.057
Relapsed	11 (14.1)	8	3	
unknown	1 (1.6)	0	1	
Overall survival (5y OS)				
Alive	56 (85.7)	22	31	***0.044***
Dead	7 (10.9)	7	2	
unknown	1 (1.6)	0	1	

^a^DNA of one patient was not available for ILT-2 rs10416697 typing, c, clinical; p, pathological; y, after neoadjuvant chemotherapy, p-values in bold italic were calculated by GraphPad Prism using two-sided Chi-square test. n.s. not significant.

### 2.2 Plasma sampling

Ethylenediaminetetraacetic acid (EDTA) blood samples were collected from the TNBC patients pre-CT at the time of first diagnosis (n = 56) and post-CT (n = 50). EDTA samples of controls and TNBC patients were processed within four hours after sampling, centrifuged at 1500 g for 10 minutes and the supernatants were stored at -80°C until usage.

### 2.3 Determination of soluble HLA-G levels

Quantification of sHLA-G plasma levels was carried out by a sandwich Enzyme-Linked ImmunoSorbent Assay (ELISA) technique (38, 41) in a 1:2 sample dilution. Purified HLA-G1 (42) was used as standard reagent in a geometric dilution starting from 30.0 to 0.117 ng/ml. Levels were determined by four-parameter curve fitting. Detection limit of sHLA-G was 0.25 ng/mL.

### 2.4 HLA-G 3’ UTR and ILT-2 rs10416697 genotyping

Cytospin preparations, containing single cell suspensions derived from mononuclear bone marrow cells of TNBC patients, were used for genomic DNA extraction by QIAamp DNA Blood Mini Kit (Qiagen, Hilden, Germany) according to manufacturer’s instructions. Typing of *HLA-G* 3’ UTR haplotype was performed by polymerase chain reaction (PCR) as previously described (39, 43). Typing of the ILT-2 rs10416697C/G SNPs located distant from ILT-2 gene promoter region was determined by a Taqman assay (ThermoFisher Scientific) according to manufacturer’s instructions. DNA samples of 163 female blood donors served as healthy control panel for HLA-G 3’ UTR and ILT-2 rs10416697 genotyping. At the time of blood sampling, the median age of HC was 50 years ranging from 25 to 71 years

### 2.5 Selection and detection of CTC

CTC were isolated from 2 x 5 ml EDTA blood by positive immunomagnetic selection using the AdnaTest EMT-2/StemCell Select™(QIAGEN GmbH, Hilden, Germany), targeting EpCAM, EGFR and HER2 as recently described (37). Briefly, labelled CTC were extracted using a magnetic particle concentrator and were lysed according to the manufacturer`s instructions. mRNA was isolated from the resulting cell lysates by oligo(dT)25-coated magnetic beads and reverse transcribed (AdnaTest EMT-2/StemCell DetectTM, QIAGEN GmbH, Hilden, Germany) with a final reaction volume of 40µl. cDNA was stored at -20°C.

A multi-marker RT-qPCR panel (QIAGEN GmbH, Hilden, Germany) was used for 46 TNBC patients pre and 44 post-CT to detect different CTC subpopulations. Genes included in the panel were AKT2, ALK, AR, AURKA, BRCA1, EGFR, ERCC1, ERBB2, ERBB3, KIT, KRT5, MET, MTOR, NOTCH1, PARP1, PIK3CA, SRC and GAPDH, respectively. Transcript-specific pre-amplification of cDNA using Multiplex PCR Master Mixes (QIAGEN GmbH, Hilden, Germany) was performed with defined PCR cycles. RT-qPCR was performed with the StepOnePlus™ (Thermo Fisher Scientific, Waltham, USA) real-time system. CTC expression data were normalized to matched expression data of HC and evaluated as described (37).

### 2.6 Statistical analysis

Continuous and categorical variables were compared using the Mann-Whitney U, Kruskal–Wallis test or two-sided Chi-square test, as appropriate. Allele and genotype frequencies of polymorphic sites as well as the contribution of haplotypes and allelic variants to clinical parameters were calculated by using two-sided Chi-square test. Receiver operating curve (ROC) analysis was performed to obtain cut-off values for categorization of continuous patient characteristics into dichotomous variables representing the optimal separation of survival curve by using the BIAS 11.10 software program (http://www.bias-online.de/). Probabilities of PFS or OS, respectively, were analyzed using Kaplan-Meier method in combination with the log-rank test implemented in the R package survminer (version 0.4.0; https://CRAN.R-project.or/package=survminer). Starting points were time points of diagnosis (first blood collection) and endpoints were death from BC or disease progression. Stepwise multivariate Cox regression according to proportional hazards assumption was used to identify prognostic factors for PFS or OS. Unless otherwise indicated, all statistical analyses have been carried out by using SPSS 25.0 software (SPSS Inc., Chicago, IL, USA) or GraphPad Prism V8.4 software (GraphPad Software, San Diego, CA, USA). *p*-values <0.05 were considered statistically significant.

## 3 Results

### 3.1 sHLA-G plasma levels are increased in TNBC patients post-CT

**Figure 1:**
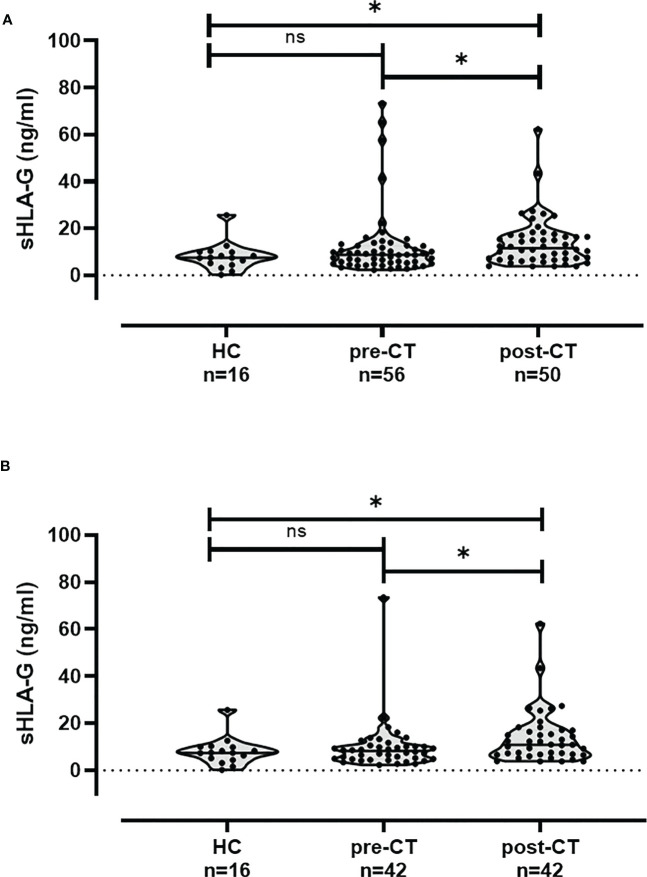
sHLA-G plasma levels are increased in TNBC patients post-CT. **(A)** sHLA-G levels in TNBC patients pre-CT compared to healthy controls (HC) did not differ, but sHLA-G levels post-CT were significantly increased compared to both groups. **(B)** Similar results were obtained by the comparison of pre- and post-CT sHLA-G levels of paired plasma samples from 42 TNBC patients. * p-value <0.05. ns: not significant.

Plasma levels of sHLA-G are given as median (range) ng/ml. The sHLA-G levels did not differ between healthy females [7.4 (0.2 – 25.7); n = 16] and TNBC patients pre-CT [8.6 (2.2 – 73.5); n=56]. However post-CT, sHLA-G levels were found to be significantly increased [11.6 (3.8 – 58.5); n = 50] in TNBC patients compared to levels of patients pre-CT (p = 0.02) or controls (p = 0.01; **Figure 1A**). Similar results were obtained by the comparison of pre- and post-CT sHLA-G levels of paired plasma samples from 42 TNBC patients (**Figure 1B**).

### 3.2 Increased sHLA-G plasma levels post-CT are associated to detrimental clinical profile in TNBC patients

**Figure 2:**
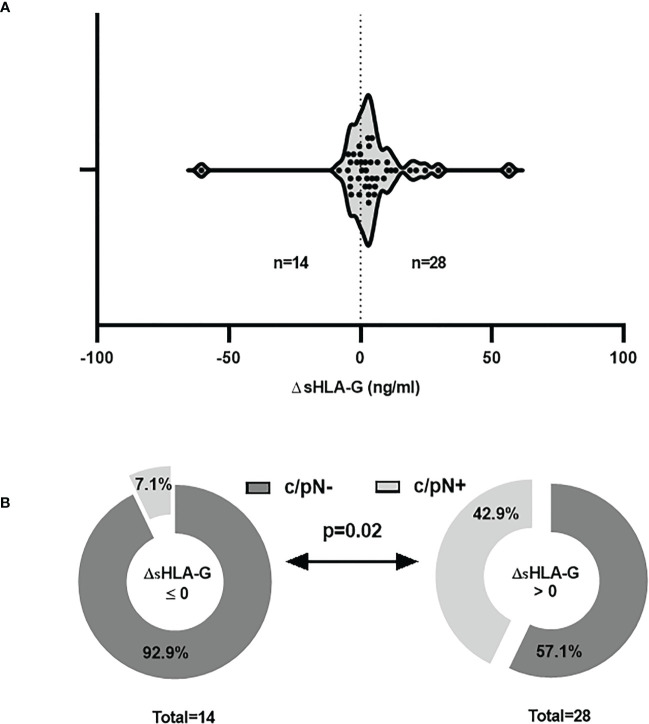
Increased sHLA-G plasma levels post-CT are associated to positive nodal status of TNBC patients at time of diagnosis. **(A)** In paired plasma samples the differences of pre- and post-CT sHLA-G levels (Δ sHLA-G) revealed a clear increase of sHLA-G levels in 28 and a reduction in 14 patients post-CT. **(B)** A higher frequency of patients with a clinical positive nodal status (c/pN+) was observed in patients with increased post-CT sHLA-G levels compared to those with reduced post-CT sHLA-G levels. Light gray or dark gray indicate patients with a clinical positive nodal status (c/pN+) and negative nodal status (c/pN-), respectively at time of diagnosis.

Overall, pre-CT (n = 56) and post-CT (n = 50) sHLA-G levels were not related to age, menopausal status of patients, histological findings, tumor burden or nodal status of TNBC patients at the time of diagnosis (**Table 1**). Similar results were obtained for pre and post CT plasma samples derived from the same patient (n = 42, Additional File 1). Regarding the differences of pre- and post-CT sHLA-G levels (Δ sHLA-G) in paired samples, a clear increase of sHLA-G levels was observed in 28 out of 42 patients post-CT, whereas only 14 patients presented a reduction of sHLA-G levels post-CT (**Figure 2A**). The increase of the sHLA-G levels after CT was significantly related to patients with a positive nodal status at the time of diagnosis (p = 0.02, **Figure 2B**). Concerning the amount of post-CT sHLA-G levels (**Table 1**), TNBC patients who developed distant metastases post-CT displayed significantly (p = 0.03) higher sHLA-G levels [17.9 (6.8 – 64.7); n = 6] than patients who did not develop distant metastases [10.7 (3.8 – 43.4); n = 44]. Patients with paired samples (Additional File 1) were found to have similar post-CT sHLA-G levels as unpaired samples, but with no significant difference (p = 0.061).

**Figure 3:**
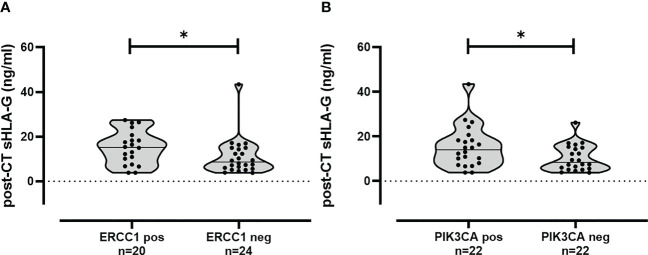
Association of increased sHLA-G levels in TNBC patients post-CT having ERCC1 and PIK3CA positive CTC subpopulation. Post-CT sHLA-G levels were increased in patients with ERCC1 **(A)** or PIK3CA **(B)** positive CTC subtypes compared to the levels in patients not expressing these CTC subtypes. Statistical significance was determined by Mann–Whitney test. * p-value <0.05.

As sHLA-G possesses tumor-supporting properties, pre- and post-CT levels were associated with the presence or absence of specific CTC subtypes. For the pre-CT situation, no association could be established between sHLA-G levels and the occurrence of a particular CTC subtype. For the post-CT situation, sHLA-G levels were significantly increased (p < 0.05) in patients harboring ERCC1- [15.2 (3.8 – 27.4); n = 20] or PIK3CA- [14.0 (3.8 – 43.4); n = 22] positive CTC compared to the levels in patients not expressing ERCC1 [8.7 (3.8 – 43.4); n = 24] or PIK3CA [8.4 (3.8 – 26.2); n = 22], respectively (**Figures 3A, B**).

### 3.3 High sHLA-G plasma levels status post-CT is a prognostic co-variate for poor PFS and OS of TNBC patients

**Figure 4:**
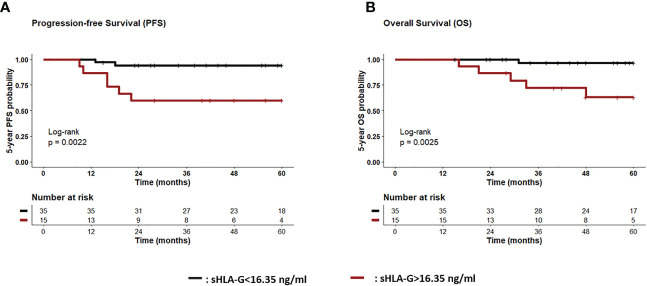
Association of high sHLA-G levels post-CT with reduced progression-free and Overall survival. Patients were divided into two groups according to cut-off level (<16.35 ng/ml>) post-CT. Kaplan-Meier plot analysis combined with log-rank test revealed that TNBC patients with sHLA-G status > 16.35 ng/mL (brown line) had a significantly reduced 5-year probability of PFS [p = 0.0022, **(A)** and OS (p = 0.0025, **(B)** compared to patients with sHLA-G status <16.35 ng/ml (black line) post-CT. Tables under Kaplan–Meier plots show corresponding numbers at risk.

To define the best threshold value regarding the prediction of 5-year PFS and OS of TNBC patients, sHLA-G post-CT (n = 50) were subjected to ROC analysis. An optimal cutoff value of 16.35 ng/ml was defined (Additional File 2A, B) for the prediction of probability of both, the PFS (sensitivity: 75%; specificity: 79%; AUC: 0.69, p = 0.08) and the OS (sensitivity: 83%; specificity: 77%; AUC: 0.77, p = 0.03). Kaplan–Meier curve analysis combined with log-rank test showed that TNBC patients with sHLA-G status > 16.35 ng/mL had a significantly reduced 5-year probability of PFS [p = 0.0022, log-rank Hazard Ratio (HR): 8.1, 95% Confidence interval (CI): 1.7–38.7] and of OS (p = 0.0025, HR: 12.9, 95% CI: 2.2–76.7) compared to patients with sHLA-G levels below this threshold value (**Figures 4A, B**).

### 3.4 HLA-G 3’ UTR haplotypes are not associated to sHLA-G plasma levels or disease status/outcome of TNBC patients

As polymorphisms in the HLA-G 3’ UTR region are reported to affect the magnitude of protein expression, 15 single nucleotide polymorphisms of the *HLA-G* 3’ UTR and haplotypes were determined in 63 TNBC patients and 163 female controls. Haplotype analysis revealed eight haplotypes with a frequency >1%. The distribution of haplotypes as well as genotypes (Additional File 3 and 4) were similar among TNBC patients and HC. HLA-G 3’ UTR haplotypes were not related to the pre- and post-CT sHLA-G levels of TNBC patients. Furthermore, no association of any HLA-G 3’ UTR haplotype with the disease status, presence of a certain CTC subtypes, therapy or disease outcome was observed (data not shown).

### 3.5 ILT-2 rs10416697C allele of the sHLA-G-related receptor is associated to detrimental clinical profile in TNBC patients

**Figure 5:**
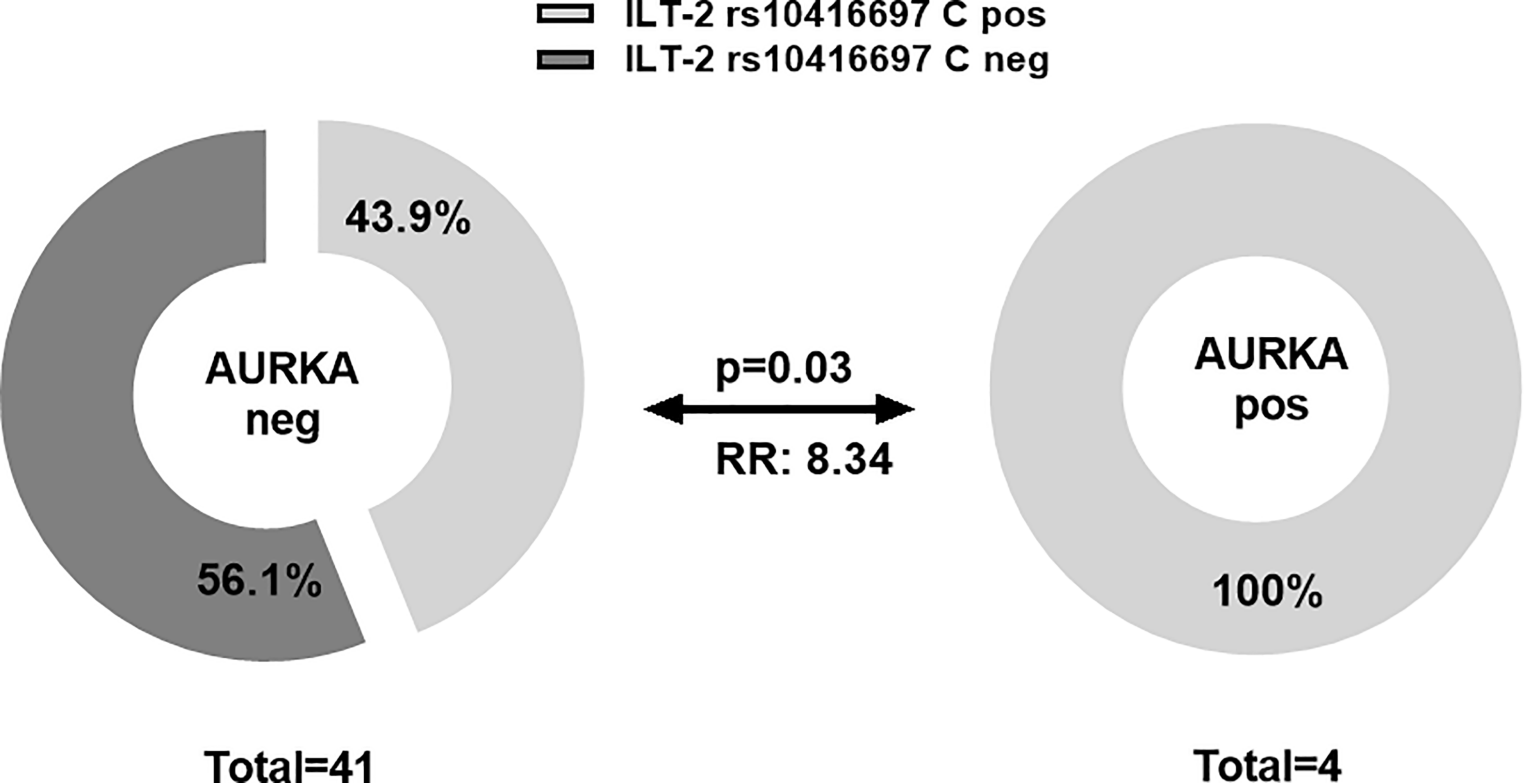
Association of ILT-2 rs10416697C allele of the sHLA-G-cognate receptor with the presence of AURKA-positive CTC post-CT. A higher frequency of patients with ILT-2 rs10416697C allele was observed in patients with AURKA-positive CTC compared to patients not expressing this CTC subtype. Light gray or dark gray indicates frequencies of ILT-2 rs10416697C positive and ILT-2 rs10416697C negative patients, respectively. RR: relative risk.

To establish a relationship between sHLA-G and its cognate receptor ILT-2, the allelic variations of SNP rs10416697 C/G in the distal ILT-2 promoter region were investigated in 63 TNBC patients and 163 female controls, as this SNP is believed to contribute to the regulation of ILT-2 expression. Allele, phenotype and genotype distribution of ILT 2 rs10416697 C/G variants (Additional File 3 and 4) were nearly identical between TNBC patients and HC. However, the frequency of the ILT 2 rs10416697C variant was significantly increased in TNBC patients who developed distant metastases post-CT (7 out of 9, p = 0.046), compared to patients without any evidence of distant metastases (2 out of 9) during follow-up time (p = 0.044; RR: 1.87, 95%CI: 1.02 – 2.87; **Table 2**). Accordingly, an increased mortality was observed in patients carrying the ILT 2 rs10416697C variant (p = 0.044, **Table 2**). Regarding CTC subtypes, the ILT-2 rs10416697C allele was significantly (p = 0.03) associated with the presence of AURKA-positive CTC (RR: 8.34; 95% CI: 0.47 to infinity; **Figure 5**) post-CT. No other CTC subpopulation could be associated with this allele pre- or post-CT.

### 3.6 ILT-2 rs10416697C allele of the sHLA-G-related receptor is a prognostic co-variate for poor PFS and OS of TNBC patients

**Figure 6:**
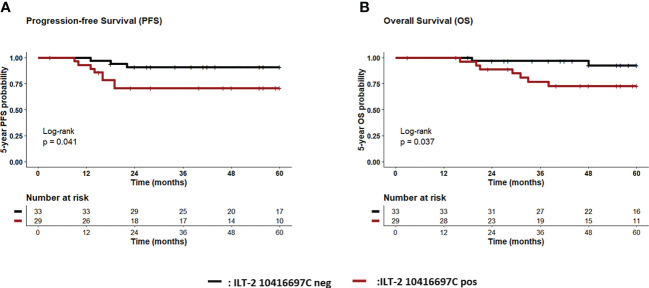
Association of ILT-2 rs10416697C allele of the sHLA-G-cognate with reduced probabilities of progression-free and overall survival of TNBC patients. Patients were divided into two groups according to their ILT-2 rs10416697C allele status. Kaplan-Meier plot analysis combined with log-rank test revealed that TNBC patients carrying the ILT-2 rs10416697C allele (brown line) had a significantly reduced 5-year probability of PFS **(A)** and OS **(B)** compared to patients being ILT-2 rs10416697C negative (black line). Tables under Kaplan–Meier plots show corresponding numbers at risk. PFS: progression-free survival, OS: overall survival.

With regard to disease outcome, Kaplan-Meier probabilities of PFS (p=0.04; HR: 3.6, 95% CI: 1.1 – 11.9) were significantly reduced for TNBC patients carrying the ILT-2 rs10416697C allele compared to patients being ILT-2 rs10416697C negative (**Figure 6A**). Correspondingly, a deteriorated OS probability was observed for ILT-2 rs10416697C allele carriers in comparison to ILT-2 rs10416697 C negative TNBC patients (p = 0.037, HR: 4.6, 95% CI: 1.2 – 17.1; **Figure 6B**).

### 3.7 High sHLA-G plasma levels status and ILT-2 rs10416697C allele of the sHLA-G-related receptor are independent prognostic co-variates for PFS and OS of TNBC patients

**Figure 7:**
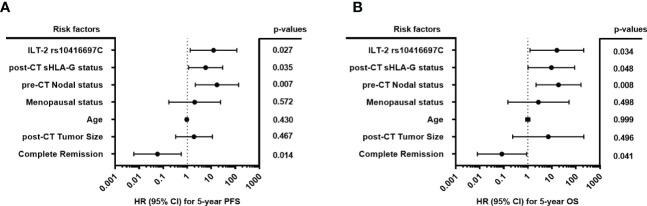
Forest plot of risk factors for progression-free and overall survival of TNBC patiernts. The forest plots visualize the multivariate analyses of the following co-variates for 5-year progression-free survival **(A)** and overall survival **(B)**: ILT-2 rs10416697C allele status, post-CT sHLA-G status (<16.35 ng/ml>), pre-CT positive nodal status (pN- vs. pN+), the menopausal status (premeno/peri vs. postmenopausal), the age (<60>), post-CT tumor size (≤T1>), and the pathological response of therapy (pCR vs. pPR/NR). 95% CI: 95% confidence interval; HR, hazard ratio; PFS: progression-free survival, OS: overall survival.

The post-CT sHLA-G status (<16.35> ng/ml), the ILT-2 rs10416697C carrier status, the pre-CT nodal status (pN- vs. pN+), the menopausal status (premeno/peri vs. postmenopausal), the age (<60>), post-CT tumor size (T1>), and the pathological response of therapy (pCR vs. pPR/NR) were subjected as co-variates to the multivariate analysis for PFS and OS. Both, the post-CT sHLA-G status >16.35 ng/ml (p = 0.027, HR: 5.8, 95%CI: 1.1 – 29.8) and the ILT-2 rs10416697C carrier status (p = 0.027, HR: 12.3, 95% CI: 1.3 – 115.3) were identified as independent risk factors for 5-year PFS (**Figure 7A**) besides a positive nodal status pre-CT (p = 0.007, HR:17.2, 95% CI: 2.1 – 136.9), whereas complete response towards CT was found to be an independent indicator (p = 0.014, HR: 0.06, 95% CI: 0.0 – 0.6) for an improved PFS. Similar results were obtained for 5-year OS (**Figure 7B**): A positive nodal status pre-CT (p = 0.008, HR: 18.8, 95% CI: 2.2 – 161.8), post-CT sHLA-G status >16.35 ng/ml (p = 0.048, HR: 9.5, 95% CI: 1.0 – 88.9) and a ILT-2 rs10416697C carrier status (p = 0.034, HR: 16.1, 95% CI: 1.2 – 211.0) were independent predictors for a poor OS, while pCR predicted a beneficial disease outcome for TNBC patients in terms of OS (p = 0.008, HR: 0.1, 95% CI: 0.0 – 0.9).

### 3.8 The combination of high sHLA-G plasma levels post-CT and ILT-2 rs10416697C allele carrier status identifies TNBC patients with the poorest PFS and OS

**Figure 8:**
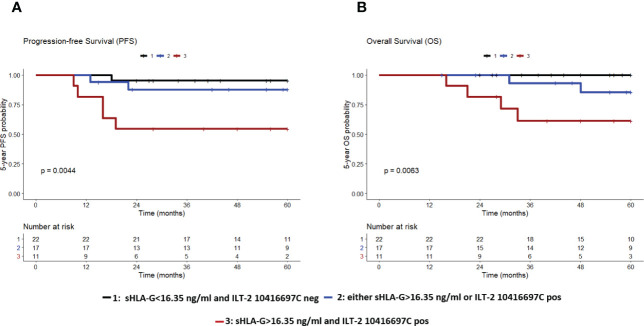
The combination of high sHLA-G plasma levels post-CT and ILT-2 rs10416697C allele carrier status identifies TNBC patients with the poorest PFS and OS. Patients were divided into three groups: Group 1 (black), comprising patients with post-CT sHLA-G levels <16.35 ng/ml and ILT-2 rs10416697C negative status; group 2 (blue), comprising patients with either post-CT sHLA-G > 16.35ng/ml or with ILT-2 rs10416697C positive status; group 3 (brown), comprising patients post-CT sHLA-G > 16.35ng/ml and with ILT-2 rs10416697C positive status. Kaplan–Meier curve of 5-year progression-free survival **(A)** and overall survival probability **(B)** combined with Mantel–Cox log-rank test revealed significantly different PFS and OS probabilities among these groups. Tables under Kaplan–Meier plots show corresponding numbers at risk.

To investigate the interaction of sHLA-G and ILT-2 rs10416697 C/G gene promoter polymorphism, patients were divided into three groups: a group of patients with a post-CT sHLA-G<16.35 ng/ml and a negative ILT-2 rs10416697C status (group 1, n=22), a group of patients with either high post-CT sHLA-G or positive ILT-2 rs10416697C status (group 2, n=17), and a group of patients with high post-CT-sHLA-G and positive ILT-2 rs10416697C status (group 3, n=11). These patients’ groups presented different PFS and OS probabilities (p=0.004 and p=0.006, respectively; **Figures 8A, B**). For PFS, multiple comparison by Peto-Pike log-rank test revealed that patients with high post-CT sHLA-G plasma levels and a positive ILT-2 rs10416697C status displayed the poorest 5-year PFS probability compared to the patient group 1 (sHLA-G<16.35 ng/ml and ILT-2 10416697C neg) with a HR of 12.5 (95% CI: 2.3 – 66.1; p=0.003, pcorr=0.009) and group 2 (either sHLA-G>16.35 ng/ml or ILT-2 10416697C pos) with a HR of 4.6 (95% CI: 1.0 – 20.4; p=0.044, pcorr=0.089). Group 1 and 2 were not significantly different in terms of PFS probability (HR: 2.7, 95% CI: 0.3 – 27.6, p=0.385, pcorr=0.385). For 5-year OS, multiple comparison by Peto-Pike log-rank test could not be performed as in patient group 1 all patients were still alive at the time of the last follow-up.

**Figure 9:**
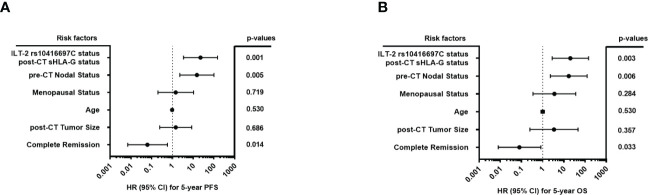
Forest plot of risk factors for progression-free and overall survival of TNBC patients with the combination of high sHLA-G plasma levels post-CT and ILT-2 rs10416697C carrier status as covariate. The forest plots visualize the multivariate analyses of the following co-variates for 5-year progression-free survival **(A)** and overall survival **(B)**: Combination of ILT-2 rs10416697C positive and post-CT, sHLA-G status >16.35 versus remaining patients, pre-CT positive nodal status (pN- vs. pN+), the menopausal status (premeno/peri vs. postmenopausal), the age (<60>), post-CT tumor size (≤T1>), and the pathological response of therapy (pCR vs. pPR/NR). 95% CI: 95% confidence interval; HR, hazard ratio; PFS: progression-free survival, OS: overall survival.

**Figure 10:**
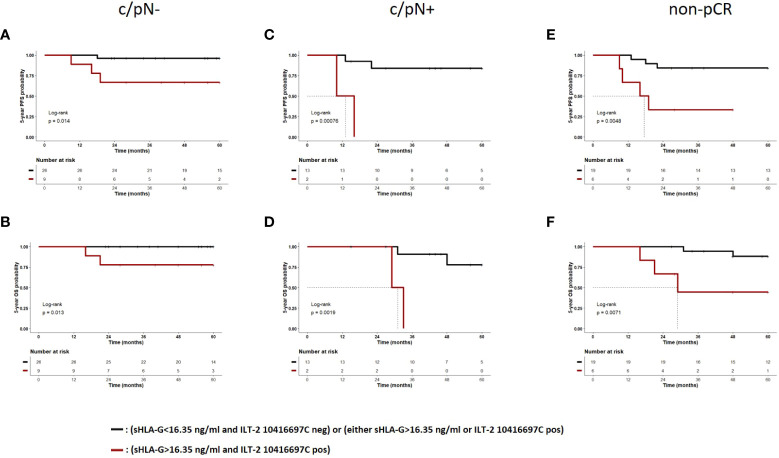
The combination of high sHLA-G plasma levels post-CT and ILT-2 rs10416697C allele carrier status identifies TNBC patients with the poorest PFS and OS. TNBC patients were divided into two groups, group 1 (black) comprising patients with no (post-CT sHLA-G<16.35 ng/ml and ILT-2 10416697C negative status) or one risk factor (either post-CT sHLA-G>16.35 ng/ml or ILT-2 10416697C positive status) of the HLA-G/ILT-2 ligand receptor axis and group 2 (brown) comprising patients with two risk factors of the HLA-G/ILT-2 ligand receptor axis (post-CT sHLA-G>16.35 ng/ml and ILT-2 10416697C positive status). Kaplan-Meier plot analysis combined with log-rank test revealed for TNBC patients with a negative pre-CT nodal status **(A, B)**, for patients with a positive pre-CT nodal status **(C, D)** and for patients with non-complete CT response **(E, F)** a significantly reduced 5-year probability of PFS and OS in TNBC patients having two risk factors of the of the HLA-G/ILT-2 ligand receptor axis compared to patients with no or only one risk factor of this axis. Tables under Kaplan–Meier plots show corresponding numbers at risk. PFS, progression-free survival; OS, overall survival; pN-, pre-CT negative nodal status; pN+, pre-CT negative nodal status; non-pCR, non-complete CT responder.

Because the 5-year PFS and OS probabilities of patients with no (group 1) or only one risk factor (group 2) of the HLA-G/ILT-2 ligand-receptor axis were very similar, these patients were grouped together and compared with patients who had both risk factors (group 3) in the multivariate analysis. All other parameters have been retained in this analysis. In terms of PFS, the prognostic value of the combined covariates (sHLA-G>16.35 ng/ml and ILT-2 rs10416697C allele carrier status) with an HR of 22.5 (95% CI: 3.4 – 148.4, p=0.001) was an even better independent indicator than a positive pre-CT node status (HR: 15.1, 95% CI: 2.3 – 100.3, p=0.005, **Figure 9A**). Similar results were obtained for OS (**Figure 9B**). Accordingly, this marker combination allowed the identification of patients with high risk of early progression/death in both, in patients with negative and in patients with positive nodal status pre-CT: node negative patients with sHLA-G>16.35 ng/ml and a ILT-2 rs10416697C allele carrier status showed a significantly reduced likelihood of PFS (p = 0.014, HR: 10.0, 95% CI: 1.0 – 102.3) and OS (p = 0.013, HR: undefined) than node negative patients with no or only one additional risk factor of the HLA-G/ILT-2 ligand receptor axis (**Figures 10A, B**). These differences were even more pronounced in patients with positive pre-CT node status. Patients with sHLA-G>16.35 ng/ml post-CT and ILT-2 rs10416697C allele carrier status revealed a significantly reduced PFS (p=0.0008, HR: 12.9, 95% CI: 0.3–570) and OS (p=0.002, HR: 11.2.95% CI: 0.3-398) with a median survival of 13 months and 31 months, respectively, compared with patients with no/one HLA-G-associated risk factor (**Figures 10C, D**).

In terms of pathological response to therapy, the stratification of TNBC patients with complete response by the number of HLA-G-related risk factors did not result in significant differences in PFS or OS (data not shown). However, in non-complete responders a marked inferior PFS (p = 0.0048, HR: 6.4, 95% CI: 0.9 – 45.8) and OS (p = 0.0071, HR: 7.7, 95% CI: 0.7 – 84.0) were observed with median survivals of 18 months and 29 months, respectively, in patients with sHLA-G>16.35 ng/ml post-CT and ILT-2 rs10416697C allele carrier status compared to patients with no/one HLA-G-associated risk factor (**Figures 10E, F**).

## 4 Discussion

Our study addresses the IC HLA-G-ILT2 ligand-receptor-axis as a possible target to identify patients at high risk of recurrence and to introduce further therapeutic targets. To follow these aspects, we determined soluble HLA-G levels pre- and post-CT, performed HLA-G 3’ UTR genotyping and determined the molecular variant of the distal gene promoter region (rs10416697) of the corresponding ILT-2 receptor and identified CTC subtypes and evaluated those parameters in context with clinical parameters.

We could demonstrate that (i) sHLA-G levels did not differ between HC and TNBC patients pre-CT, but increased sHLA-G levels were found post-CT being associated with ERCC1 or PIK3CA-CTC subtypes and with deterioration of disease outcome in univariate and multivariate analysis; (ii) HLA-G 3’ UTR genotypes were not related to disease outcome but (iii) the molecular variant rs10416697C of the distal gene promoter region of the HLA-G cognit receptor ILT-2 is associated with AURKA-positive CTC and with disease outcome in uni- und multivariate analysis. Importantly, (iv) the combination of high post-CT sHLA-G plasma levels and ILT-2 rs10416697C allele carrier status identifies TNBC patients at high risk of relapse, both in patients with negative nodal status and in patients with positive nodal status before CT, and in patients who do not respond completely to therapy, i.e., in subgroups of patients who urgently require new therapeutic strategies due to prognostically poor disease outcome.

Our cohort of TNBC patients thus presents a different picture from previous studies of locally advanced BC patients, in whom significantly elevated sHLA levels were found before CT compared with HC (38). Furthermore, post-CT sHLA-G levels, rather than pre-CT HLA-G levels, are of prognostic significance for disease progression in TNBC. Because estrogen receptor expression in locally advanced BC patients was associated with elevated non-vesicular HLA-G levels before CT (38) and functional studies also demonstrated that progesterone can stimulate HLA-G gene expression, the difference in levels and their prognostic relevance in these two BC cohorts may be explained in part by the absence of hormone receptors in TNBC and their presence in the majority of locally advanced BC patients (34, 44, 45). Furthermore, in breast cancer tissues HLA-G expression was found to be positively correlated with the hormone receptors expression and slightly increased sHLA-G plasma levels were observed in patients with positive hormone receptors compared to those without hormone receptor expression (34). In accordance with our study, a former study on TNBC patients also observed similar sHLA-G levels in patients and HC (46). Thus, the lack of hormone receptor expression may explain why sHLA-G levels in TNBC patients before CT are within the range of levels in HC. Concerning the histological subtypes of our patients’ group, no significant association of pre- and post-CT sHLA-G levels with the histological subtypes was observed. Contrarily to our current study and to the study by He et al. (34) another study showed significantly increased sHLA-G levels in patients having a mixed type of ductal/lobular carcinoma compared to pure ductal or lobular carcinoma. Unfortunately, this study did not provide data on hormone receptor expression to allow a direct comparison between these three studies (47).

The increase in sHLA-G levels after CT was significantly related to lymph nodes affected at diagnosis, which may release HLA-G molecules under CT. Here, one would not expect chemotherapy to stimulate the HLA-G expression; rather, therapy-induced cell destruction is responsible for the HLA-G release. However, this would imply that these cells had high levels of HLA-G. The immunobiological relevance of high sHLA-G levels after CT is supported by the association with distant metastases that developed during the course of the disease. Interestingly, a recent *in vitro* study provided strong evidence that the presence of sHLA-G prior to T cell activation leads to an increase in ILT-2 expression as well as co-expression of other immune checkpoints such as CTLA4, PD-1, and CD95 on CD8+ T cells, suggesting a distinct immunosuppressive/exhausted phenotype of CD8+ T cells, thereby potentially subverting tumor cell immune surveillance (48).

Regulation of HLA-G expression involves posttranscriptional processes, in which nucleotide variability at the 3’ UTR can alter HLA-G mRNA stability or microRNA (32, 49). The significance of HLA-G 3’ UTR SNP or their combination as haplotypes for therapy or disease progression in BC patients has been investigated in several studies (39, 50–55). However, studies that included only TNBC patients have not been conducted. In contrast to our previous study, HLA-G 3’ UTR haplotypes are not associated with therapy or disease outcome in TNBC, which again may be due to the underlying TNBC-specific biology or to chemotherapy rather than targeted therapies for TNBC.

The pCR is a surrogate marker for improved PFS and OS (8, 9) in BC patients and therefore, a primary endpoint in clinical trials, leading to approval of new therapies. In our cohort, we could confirm that pCR was an independent factor predicting a beneficial disease outcome in terms PFS and OS. Furthermore, a positive nodal status pre-CT was identified as an independent risk factor for 5-year PFS and OS, congruent to literature (56, 57).

Identification of sHLA-G after CT as an independent prognostic marker in early-stage TNBC patients helps to identify patients at high risk of recurrence probability. Importantly, the molecular variant rs10416697C of the distal ILT-2 gene promoter region, which is a regulative element for the expression of the cognate HLA-G receptor ILT-2 (33), is associated with disease progression and OS in TNBC patients in univariate and multivariate analysis. In this context it is of interest that a previous study demonstrated an overexpression of ILT-2 receptor on NK cells, which is associated to sHLA-G levels and an impaired NK cell function in TNBC patients. Moreover, in-vitro experiments revealed that the NK cell activity can be restored by functional blocking of ILT-2 receptor (46).

With regard to the CTC subpopulation, a clear association of ERCC1- and PIK3CA-positive CTC with high sHLA-G levels and an association of the ILT-2 rs10416697C allele with the presence of AURKA-positive CTC were observed after CT. A previous study with the same cohort of patients has already shown that TNBC-derived CTCs upregulate a large number of the genes studied or maintain their expression frequency at a high level after CT. This included all genes related to the PIK3CA pathway, all resistance-related genes (BRCA1, AURKA, ERCC1), and ERBB3. Interestingly, platinum-based therapy was associated with a shortened PFS and also correlated with CTC-PIK3CA overexpression after but not before therapy, likely explaining the expression of genes related to resistance (37).

Consequently, the resistant CTC subtypes and the presence of the molecular variant rs10416697C of the distal gene promoter region of the HLA-G probably cognate receptor ILT-2 display different qualities in predicting the course of disease, which should both be implemented in risk management of TNBC patients. Moreover, the combination of high sHLA-G plasma levels post-CT and ILT-2 rs10416697C allele carrier status with positive nodal status and non-pCR, a subgroup of patients with worst outcome and with urgent need for new therapeutic strategies could be identified in our trial. Here, targeting the HLA-G-ILT2 ligand-receptor axis seems to be a promising tool for the immunotherapeutic intervention in TNBC patients. In preclinical trials, blocking the IC HLA-G-ILT2 ligand-receptor-axis by a first-in human ILT2 blocking antibody (BND-22) has shown efficient antitumor activity (52). Recently, a phase 1/2 trial in advanced or metastatic solid tumors, including them non-small cell lung cancer, cervical, colorectal and BC, evaluates NGM707, a novel dual antagonist antibody that inhibits the ILT2 and ILT4, as a monotherapy or in combination with Pembrolizumab (ClinicalTrials.gov Identifier: NCT04913337).

Conclusion: In our study, we were able to perform risk stratification based on a stable molecular genetic marker, a clinical parameter at baseline, and follow-up parameters influenced by therapy. Positive ILT-2 rs10416697C carrier status at baseline, positive nodal status before CT, and response to therapy after CT and sHLA-G status (>16.35 ng/ml) after CT were independent prognostic markers of worse 5-year PFS and OS in our cohort of early-stage TNBC patients. The combination of these markers identified patients with the highest risk of recurrence and greatest need for new therapeutic strategies: (i) ILT-2 rs10416697C positive carrier status, and sHLA-G status (>16.35 ng/ml) after CT and positive nodal status before CT, (ii) ILT-2 rs10416697C positive carrier status and sHLA-G status (>16.35 ng/ml) after CT and non-pathological complete response. In addition, certain CTC subpopulations after CT were significantly associated with ILT-2 rs10416697C allele (AURKA) and significantly elevated sHLA-G levels (ERCC1 or PIK3CA) after CT as independent prognostic markers for outcomes.

Nevertheless, the clear limitation of our study is the small patient cohort, as well as the fact that it is a single-center study, which on the other hand is an advantage as the same protocol was applied. In this patient cohort none of the patients was treated with immunotherapy as it wasn’t the standard of care at that time.

## Data availability statement

The original contributions presented in the study are included in the article/**Supplementary Material**, further inquiries can be directed to the corresponding author/s.

## Ethics statement

The studies involving human participants were reviewed and approved by Clinical ethic committee of the University Hospital Essen (05/2856). The patients/participants provided their written informed consent to participate in this study.

## Author contributions

OH, A-KB, SK-B, VR: Conceived and designed research, performed the experiments, interpreted data, performed statistical analysis, wrote the initial draft and read and approved the final article. SW, SS, JH, ES: Performed experiments. RK, PH: Interpreted data and read and approved the final article. A-KB, HR; RK, OH: Collected and provided clinical data, interpreted data, read and approved the final article. All authors contributed to the article and approved the submitted version.
