# Supplementary material for: Elevated sHLA-G plasma levels post chemotherapy combined with ILT-2 rs10416697C allele status of the sHLA-G-related receptor predict poorest disease outcome in early triple-negative breast cancer patients
Source: Front Immunol. 2023 May 22;14:1188030. doi: 10.3389/fimmu.2023.1188030 (PMC10239857; doi:10.3389/fimmu.2023.1188030)
Supplement: Supplementary file 1 [file DataSheet_1.docx]

**Additional File 1: Patients’ characteristics and their association of pre and post sHLA-G levels of paired TNBC patients**

| Parameter | **Pre-CT HLA-G (ng/ml)** | | | | **Post-CT HLA-G (ng/ml)** | | |
| --- | --- | --- | --- | --- | --- | --- | --- |
|  | n | Med. | Min | Max | Med. | Min | Max |
| **Age (years)** |  |  |  |  |  |  |  |
| >60 | 10 | 10.37 | 73.5 | 3.44 | 11.25 | 43.41 | 5.1 |
| <60 | 32 | 7.17 | 22.26 | 2.23 | 10.88 | 64.71 | 3.79 |
| **Menopausal Status** |  |  |  |  |  |  |  |
| Premenopausal | 8 | 7.53 | 18.28 | 2.23 | 8.81 | 14.96 | 6.02 |
| Perimenopausal | 9 | 6.53 | 16.08 | 3.47 | 12.36 | 25.34 | 3.81 |
| Postmenopausal | 25 | 8.59 | 73.5 | 2.75 | 10.65 | 64.71 | 3.79 |
| **Histology** |  |  |  |  |  |  |  |
| Ductal | 28 | 8.97 | 73.5 | 2.7 | 12.35 | 64.71 | 3.81 |
| Lobular | 1 | 12.67 | 12.67 | 12.67 | 18.28 | 18.28 | 18.28 |
| Others | 11 | 4.89 | 9.6 | 2.23 | 7.37 | 27.41 | 3.79 |
| Unknown | 2 | 8.94 | 10.6 | 7.28 | 12.24 | 20.67 | 3.81 |
| **Tumor grading** |  |  |  |  |  |  |  |
| I | 0 | . | . | . | . | . | . |
| II | 9 | 7.05 | 11.73 | 4.28 | 14.96 | 25.34 | 3.81 |
| III | 33 | 8.59 | 73.5 | 2.23 | 10.65 | 64.71 | 3.79 |
| unknown | 0 | . | . | . |  |  |  |
| **Ki 67** |  |  |  |  |  |  |  |
| 0-10% | 2 | 8.44 | 9.6 | 7.28 | 5.16 | 17.02 | 3.81 |
| 11-30% | 3 | 6.45 | 13.92 | 4.28 | 10.79 | 64.71 | 3.79 |
| >30% | 30 | 7.98 | 73.5 | 2.23 | 12.34 | 43.41 | 6.02 |
| unknown | 7 | 9.78 | 13.81 | 4.42 | 5.16 | 17.02 | 3.81 |
| **Tumor size at diagnosis (c/pT)** |  |  |  |  |  |  |  |
| c/p T1a-c | 19 | 6.45 | 22.26 | 2.7 | 12.22 | 64.71 | 3.79 |
| c/p T2 | 21 | 8.59 | 18.28 | 2.23 | 10.65 | 25.34 | 3.81 |
| c/p T3 | 2 | 41.81 | 73.5 | 10.12 | 10.28 | 13.05 | 7.51 |
| c/p T4 | 0 | . | . | . | 0 | . | . |
| **Tumor size after CT (ypT)** |  |  |  |  |  |  |  |
| ypT0 | 20 | 8.58 | 22.26 | 2.23 | 9.96 | 64.71 | 3.79 |
| ypT1 | 13 | 7.9 | 18.28 | 2.7 | 10.1 | 43.41 | 3.81 |
| ypT2 | 7 | 9.6 | 73.5 | 3.44 | 16.38 | 25.34 | 6.77 |
| ypT3 | 1 | 4.89 | 4.89 | 4.89 | 7.37 | 7.37 | 7.37 |
| ypT4 | 1 | 5.24 | 5.24 | 5.24 | 26.46 | 26.46 | 26.46 |
| unknown | 0 |  |  |  |  |  |  |
| **Nodal Status at diagnosis (c/pN)** |  |  |  |  |  |  |  |
| Node-negative (c/pN-) | 29 | 8.56 | 73.5 | 2.23 | 9.45 | 43.41 | 3.79 |
| Node-positive (c/pN+) | 13 | 5.84 | 16.08 | 3.44 | 12.36 | 64.71 | 6.77 |
| **Nodal status after CT (ypN)** |  |  |  |  |  |  |  |
| Node-negative (ypN-) | 2 | 8.78 | 13.27 | 4.28 | 9.6 | 15.39 | 3.81 |
| Node-positive (ypN+) | 0 |  |  |  |  |  |  |
| unknown | 40 | 8.23 | 73.5 | 2.23 | 10.88 | 64.71 | 3.79 |
| **Chemotherapy** |  |  |  |  |  |  |  |
| Neoadjuvant | 42 | 8.23 | 73.5 | 2.23 | 11.58 | 3.79 | 64.71 |
| Adjuvant | 0 |  |  |  |  |  |  |
| unknown |  |  |  |  |  |  |  |
| **Pathological response** |  |  |  |  |  |  |  |
| Complete response | 21 | 8.56 | 22.26 | 2.23 | 10.65 | 64.71 | 3.79 |
| Partial response | 18 | 9.28 | 73.5 | 2.7 | 12.71 | 43.41 | 3.81 |
| No response | 3 | 4.89 | 7.05 | 3.44 | 7.37 | 17.16 | 6.77 |
| unknown |  |  |  |  |  |  |  |
| **Distant metastases** |  |  |  |  |  |  |  |
| yes | 5 | 6.53 | 16.08 | 3.44 | 18.28 | 64.71 | 6.77 |
| no | 37 | 8.56 | 73.5 | 2.23 | 10.65 | 43.41 | 3.79 |
| unknown | 0 |  |  |  |  |  |  |
| **Recurrence (5y-PFS)** |  |  |  |  |  |  |  |
| Alive | 35 | 8.59 | 73.5 | 2.23 | 10.65 | 43.41 | 3.79 |
| Relapsed | 7 | 7.05 | 16.08 | 3.44 | 17.16 | 64.71 | 3.81 |
| unknown | 0 |  |  |  |  |  |  |
| **Overall survival (5y OS)** |  |  |  |  |  |  |  |
| Alive | 37 | 8.56 | 73.5 | 2.23 | 10.65 | 43.41 | 3.79 |
| Dead | 5 | 6.53 | 16.08 | 3.44 | 18.28 | 64.71 | 6.77 |
| unknown | 0 |  |  |  |  |  |  |

CT: chemotherapy Med.: Median; Min: Minimum; Max: Maximum; c: clinical; p: pathological; c: before chemotherapy; y: after neoadjuvant chemotherapy;

**Additional File 2:** **A, B: Receiver operating characteristic (ROC) analyses for the threshold determination of sHLA-G levels post chemotherapy for the 5-year PFS and OS.**

Additional file 3. *HLA-G* 3’ UTR Haplotype frequencies and the *ILT-2rs10416697 allele frequencies* at the ILT-2 gene promoter region in TNBC patients (n=63) and healthy female controls (n=163)

| **Haplotype** | **Frequency**  **female HC** | **Frequency TNBC** | **p_a_** | **OR (95% CI)** |
| --- | --- | --- | --- | --- |
| **UTR-1** | 34.7 | 33.6 | 0.826 | 0.98 (0.61 – 1.45) |
| **UTR-2** | 29.5 | 31.8 | 0.648 | 1.11 (0.71 – 1.75) |
| **UTR-3** | 8.0 | 4.8 | 0.307 | 0.58 (0.24 – 1.38) |
| **UTR-4** | 15.3 | 15.1 | >0.999 | 0.98 (0.55 – 1.71) |
| **UTR-5** | 2.8 | 0.8 | 0.296 | 0.28 (0.02 – 1.72) |
| **UTR-6** |  |  |  |  |
| **UTR-7** | 4.0 | 7.9 | 0.097 | 2.07 (0.90 – 4.87) |
| **UTR-18** | 1.2 | 1.6 | 0.673 | 1.29 (0.24 – 5.63) |
| **ILT-2 rs10416697 C** | 33.7 | 28.6 | 0.314 | 0.78 (0.50 – 1.24) |
| **ILT-2 rs10416697 G** | 66.3 | 71.4 |  |  |

Haplotype phasing was assessed by PHASE 2.1 software using default parameters. Only haplotypes with frequencies >1 % were listed. TNBC – triple-negative breast cancer; HC – healthy controls; UTR – untranslated region. ^a^p-values were calculated by GraphPad Prism using two-sided Chi-square test for the evaluation of haplotypes and genotypes, alpha<0.05; OR, odds ratio

Additional File 4. Distribution of *HLA-G* 3’UTR haplotypes and ILT-2rs10416697 allele phenotypes and corresponding genotypes in TNBC patients and healthy female donors.

|  | **Female HC** | **TNBC** |  | OR  (95% CI) |
| --- | --- | --- | --- | --- |
|  | N=163 (%) | N=63 (%) | *p^a^* |  |
| **UTR-1** |  |  |  |  |
| pos | 91 (55.8) | 34 (54.0) | 0.881 | 0.92 (0.52 – 1.67) |
| neg | 72 (44.2) | 29 (46.0) |  |  |
| Genotypes |  |  |  |  |
| UTR-1/UTR-1 | 22 (13.5) | 8 (12.7) | 0.966 |  |
| UTR-1/UTR-X | 69 (43.3) | 26 (41.3) |  |  |
| UTR-X/UTR-X | 72 (44.2) | 29 (46.0) |  |  |
| **UTR-2** |  |  |  |  |
| pos | 85 (52.1) | 25 (55.6) | 0.635 | 1.22 (0.67 – 2.26) |
| neg | 78 (47.9) | 28 (44.4) |  |  |
| Genotypes |  |  |  |  |
| UTR-2/UTR-2 | 11 (6.7) | 5 (7.9) | 0.882 |  |
| UTR-2/UTR-X | 74 (45.4) | 30 (47.9) |  |  |
| UTR-X/UTR-X | 78 (47.9) | 28 (44.4) |  |  |
| **UTR-3** |  |  |  |  |
| pos | 25 (15.3) | 6 (9.5) | 0.289 | 0.58 (0.24 – 1.50) |
| neg | 138 (84.7) | 57 (90.5) |  |  |
| Genotypes |  |  |  |  |
| UTR-3/UTR-3 | 1 (0.6) | 0 (0.0) | 0.475 |  |
| UTR-3/UTR-X | 24 (14.7) | 6 (9.5) |  |  |
| UTR-X/UTR-X | 138 (84.7) | 57 (90.5) |  |  |
| **UTR-4** |  |  |  |  |
| pos | 45 (23.5) | 18 (28.6) | 0.870 | 1.05 (0.55 – 1.95) |
| neg | 118 (76.5) | 45 (71.4) |  |  |
| Genotypes |  |  |  |  |
| UTR-4/UTR-4 | 5 (3.1) | 1 (1.6) | 0.783 |  |
| UTR-4/UTR-X | 40 (24.5) | 17 (27.0) |  |  |
| UTR-X/UTR-X | 118 (76.5) | 45 (71.4) |  |  |
| **UTR-5** |  |  |  |  |
| pos | 9 (5.5) | 1 (1.6) | 0.290 | 0.28 (0.02 – 1.75) |
| neg | 154 (94.5) | 62 (98.4) |  |  |
| Genotypes |  |  |  |  |
| UTR-5/UTR-5 | 0 (0.0) | 0 (0.00) | n.a. |  |
| UTR-5/UTR-X | 9 (5.5) | 1 (1.6) |  |  |
| UTR-X/UTR-X | 154 (94.5) | 62 (98.4) |  |  |
| **UTR-6** |  |  |  |  |
| pos | 2 (1.2) | 1 (1.6) | >0.99 | 1.30 (0.09 – 11.31) |
| neg | 161 (98.8) | 62 (98.4) |  |  |
| Genotypes |  |  |  |  |
| UTR-6/UTR-6 | 0 (0.00) | 0 (0.00) | n.a. |  |
| UTR-6/UTR-X | 2 (1.2) | 1 (1.6) |  |  |
| UTR-X/UTR-X | 161 (98.8) | 62 (98.4) |  |  |
| **UTR-7** |  |  |  |  |
| pos | 12 (7.4) | 8 (12.7) | 0.203 | 1.83 (0.72 – 4.54) |
| neg | 151 (92.6) | 55 (87.3) |  |  |
| Genotypes |  |  |  |  |
| UTR-7/UTR-7 | 1 (0.6) | 2 (3.2) | 0.240 |  |
| UTR-7/UTR-X | 11 (6.7) | 6 (9.5) |  |  |
| UTR-X/UTR-X | 151 (92.6) | 55 (87.3) |  |  |
| **UTR-18** |  |  |  |  |
| pos | 4 (2.5) | 2 (3.2) | 0.672 | 1.30 (0.24 – 5.71) |
| neg | 159 (97.5) | 61 (96.8) |  |  |
| Genotypes |  |  |  |  |
| UTR-18/UTR-18 | 0 (0.00) | 0 (0.00) | n.a. |  |
| UTR-18/X | 4 (2.5) | 2 (3.2) |  |  |
| UTR-X/UTR-X | 159 (97.5) | 61 (96.8) |  |  |
| **ILT-2 rs10416697** |  |  |  |  |
| C pos | 90 (55.2) | 29 (46.0) | 0.237 | 0.69 (0.38 – 1.23) |
| C neg | 73 (44.8) | 34 (54.0) |  |  |
| Genotypes |  |  |  |  |
| CC | 20 (12.3) | 7 (11.1) | 0.454 |  |
| CG | 70 (42.9) | 22 (34.9) |  |  |
| GG | 73 (44.8) | 34 (54.0) |  |  |

CI – confidence interval; HC – healthy controls; TNBC – triple-negative breast cancer; n.a. – not applicable; neg – negative; OR – odds ratio; pos – positive; UTR – untranslated region; UTR-X – every other UTR

^a^p-values were calculated by GraphPad Prism using two-sided Chi-square test for the evaluation of haplotypes and genotypes, alpha<0.05; OD, odds ratio
